# Effects of Buyang Huanwu Decoction on Ventricular Remodeling and Differential Protein Profile in a Rat Model of Myocardial Infarction

**DOI:** 10.1155/2012/385247

**Published:** 2012-09-19

**Authors:** Ying Chun Zhou, Bin Liu, Ying Jia Li, Lin Lin Jing, Ge Wen, Jing Tang, Xin Xu, Zhi Ping Lv, Xue Gang Sun

**Affiliations:** ^1^Nanfang Hospital, Southern Medical University, Guangdong, Guangzhou 510515, China; ^2^The Key Laboratory of Molecular Biology, State Administration of Traditional Chinese Medicine, School of Traditional Chinese Medicine, Southern Medical University, Guangdong, Guangzhou 510515, China; ^3^Yuebei People's Hospital, Guangdong, Shaoguan 512026, China

## Abstract

Buyang Huanwu decoction (BYHWD) is a well-known and canonical Chinese medicine formula from “Correction on Errors in Medical Classics” in Qing dynasty. Here, we show that BYHWD could alleviate the ventricular remodeling induced by left anterior descending (LAD) artery ligation in rats. BYHWD treatment (18 g/kg/day) decreased heart weight/body weight (HW/BW), left ventricle (LV) dimension at end diastole (LVDd) and increased LV ejection fraction (LVEF) and LV fractional shortening (LVFS) significantly compared to model group at the end of 12 weeks. The collagen volume of BYHWD group was more significantly decreased than that of model group. Proteomic analysis showed that atrial natriuretic factor (ANF) was downregulated; heat shock protein beta-6 (HSPB6) and peroxiredoxin-6 (PRDX6) were upregulated in BYHWD-treated group among successfully identified proteins. The apoptotic index (AI) was reduced by BYHWD accompanied by decreased expression of Bax and caspase 3 activity, increased Bcl-2/Bax ratio, and phosphorylation of HSPB6 compared to that of model group. Taken together, these results suggest that BYHWD can alleviate ventricular remodeling induced by LAD artery ligation. The antiremodeling effects of BYHWD are conferred by decreasing AI through affecting multiple targets including increased Bcl-2/Bax ratio and decreased caspase 3 activity that might be via upregulated PRDX6, phosphorylation of HSPB6 and subsequently reduction of ANF.

## 1. Introduction

Myocardial infarction (MI) is one of the major causes of heart failure. According to latest estimates, 610,000 new attacks and 325,000 recurrent attacks of MI were reported in America annually [1]. We have been witnessing several rapid developments in the diagnosis and treatment of cardiovascular diseases. In fact, numerous patients can now survive an acute MI and undergo remodeling of their left ventricle (LV). According to left ventricular remodeling, a series of histopathologic changes occur in the myocardium. Structural changes are also observed in the left ventricular chamber as the MI diminishes through systolic performance and stroke volume [2]. The analysis between LV remodeling and clinical outcomes indicates that drug therapies contain or reverse the remodeling process. This can have favorable natural history effects in short-term and long-term therapies [3]. Traditional Chinese medicines (TCM) have protective effects on ventricular remodeling [4, 5]. These provide alternative methods on prevention ventricular remodeling to ameliorate the prognosis of MI. To elucidate the underlying mechanism of effective TCM formulae on ventricular remodeling, especially the classical formulae, would not only provide a better understanding of them, but also give a translational chance for the ancient formulae.

Buyang Huanwu decoction (BYHWD) is a well-known canonical Chinese medicine formula from the “Correction on Errors in Medical Classics.” In terms of TCM, it invigorates Qi to promote blood circulation and dredge collaterals. Since hundreds of years, it has been used to improve the neurological functional recovery of stroke-induced disabilities in China [6]. BYHWD reduces the spinal ischemia/reperfusion damage [7]. Furthermore, it ameliorates the cerebral ischemic injury [8]. It also improves the performance of neurological behavior, thereby reducing the infarction volume in ischaemic brains of rats [6]. Recently, it has been reported that BYHWD ameliorated coronary heart disease with Qi deficiency and blood stasis syndrome [9]. It also inhibited the ischemic myocardial injury [10]. The protective effects of BYHWD on ventricular remodeling have been reported in MI patients [11] and animal models [12]. However, its underlying mechanisms continue to remain elusive. A proteomics-based approach was used to identify the response of differentially expressed proteins to BYHWD treatment in ischemia-induced ventricular remodeling of rats. In the clinical application of BYHWD, this study provided more useful and theoretical information.

## 2. Materials and Methods

### 2.1. Preparation of Drugs

BYHWD is composed of Radix Astragali membranaceus (root of *Astragalus membranaceus *var.* mongholicus* (Bunge) Hsiao, Inner Mongolia, China), Radix Angelicae Sinensis (root of *Angelica sinensis *(Oliv.) Diels, Gansu, China), Radix Paeonia Rubra (root of *Paeoniae lactiflora* Pall, Sichuan, China), Rhizoma Chuanxiong (root of *Ligusticum chuanxiong* Hort., Sichuan, China), Semen Persicae (seed of *Prunus persica* (L.) Batsch, Hunan, China), Flos Carthami (flower of *Carthamus tinctorius *L., Henan, China), and Lumbricus (*Pheretima aspergillum* (Perrier), Guangdong, China). The raw herbs for BYHWD were purchased from the affiliated Nan Fang Hospital of Southern Medical University. These were mixed in the ratio of 120 : 10 : 10 : 10 : 10 : 10 : 4.5 (dry weight). Aqueous extracts of BYHWD were extracted at 80°C by stirring it for 1 h using 10 volumes of distilled water (v/m). Then, we centrifuged the extract at 1.500 × g at room temperature. To obtain the semisolid BYHED solution, the supernatant was collected and subjected to condensation under reduced pressure of 70°C [13]. The quality of BYHWD was controlled by HPLC analysis (see, supplementary material available online at doi:10.1155/2012/385247). The quality controlled BYHWD was suspended again in 0.9% saline at a final concentration of 2 g of crude drug per milliliter. The solution was stored in aliquots at −20°C.

### 2.2. Animals and Experimental Myocardial Infarction

We housed male Wistar rats, each weighing 200–250 g (specific pathogen-Free, Certificate no. 2011A036). They were provided with free access to water and rodent chow at 20 ~ 22°C through a 12 h light-dark cycle. In accordance with the guidelines of the Instituted Animal Care and Use Committee of Southern Medical University, we performed all the procedures involving laboratory animal use. Before conducting the experiment, rats were allowed to adapt with the new environment for 1 week. Rats were anesthetized by subjecting them to isoflurane inhalation. They were intubated and placed on a rodent ventilator (SAR-830, IITC, USA). We performed positive pressure artificial respiration, and the body temperature was maintained at 37°C. After performing left thoracotomy, we ligated the left large marginal coronary artery at approximately 2 mm below the left atrium using a 5-0 Ethicon silk suture. Successful ligation was confirmed by observing the pallor of left ventricular free wall and bulging of the left atrium. Sham-operated animals were subjected to an identical procedure without tying the suture.

### 2.3. Experimental Groups and Mortality

In total, 40 rats were used. 10 animals were dealt as sham controls, whereas 30 rats underwent coronary ligation. Within 48 h of occlusion, 4 animals died. A peri-infarct mortality rate of 13.33% was attained. The remaining 26 rats were randomized into the following two groups: model (*n* = 13) and BYHWD (*n* = 13). BYHWD 18 g/kg is administered by gavage once a day during the 12 weeks. Equal volume of normal saline was used for model and sham group. Five rats died before the end of the protocol (model 3; BYHWD 2).

### 2.4. Histological Analyses

Rat hearts were fixed in 4% paraformaldehyde and embedded in paraffin. Serial sections (5 *μ*m) were routinely stained using hematoxylin-eosin and Masson's trichrome staining. This was examined under a light microscope (×400) and photographed for morphological analysis. Collagen volume fraction and total LV area of each image were measured using the NIS-Elements (Nikon Instech Co., Ltd., Tokyo, Japan), and the percentage of collagen fraction was calculated using the following formula: (collagen area/total LV area) × 100.

### 2.5. Transthoracic Echocardiography Measurements

A non-invasive transthoracic echocardiography method was used to evaluate the morphology and function of left ventricle. Transthoracic echocardiographic determinations were conducted in the lateral decubitus position through a commercially available echocardiograph (Philips iU22 Ultrasound System with a 6–15 MHz transducer, Bothell, WA, USA). Echocardiography was performed in anesthetized animals using sodium pentobarbital (80 mg/kg, i.p., Fluka, Sigmal-Aldrich, Inc., St. Louis, MO, USA). It consisted of a two-dimensional mode, that is, time-motion (TM) mode and blood flow measurements in pulsed Doppler mode. TM mode measurements were performed in the parasternal long-axis view. Some of the measured parameters included diastolic septal wall thickness, diastolic posterior wall thickness of the LV, and end-diastolic and end-systolic diameter of the LV. We calculated LV shortening fraction using the following formula: [(end-diastolic diameter-end-systolic diameter)/end-diastolic diameter × 100]. According to the method described by Boissiere et al., end systolic wall stress was measured [14]. All the data was measured on digital recordings through one experimenter. To ensure correct measurement before experimentation, intra-animal and intraobserver variability were tested on 10 animals.

### 2.6. Sample Preparation and Two-Dimensional Electrophoresis

The area that was at risk in the left ventricle of rats in model, BYHWD, and sham group was removed, flash-frozen in liquid nitrogen, and stored at −80°C for use. Aliquots of heart tissue (40 mg) were homogenized in liquid nitrogen and resuspended in ice-cold lysis buffer (7 mol/L urea, 2 mol/L thiourea, 4% (w/v) CHAPS, 20 mmol/L Tris, 65 mmol/L DTT, 0.2% pharmalyte 3/10 ampholyte) supplemented with protease inhibitor cocktail tablets Complete Mini (Roche Diagnostics, Basel, Switzerland). The lysate was centrifuged at 15,000 × g for 30 min at 4°C. The supernatant protein concentration was determined using the PlusOne 2-D Quant kit (GE Healthcare Bio-Science, Uppsala, Sweden). We took out and mixed about 2 mg protein per sample in the same group. Samples containing protein (300 *μ*g) were loaded in each tube through an isoelectric focusing (IEF) system (IPGphor II, GE). Precast: 24 cm, pH: 3–10 IPG gel strips, and isoelectrofocusing for the total Vh was 70 kVh. The gel strips were equilibrated and transferred to the second-dimension gels (10% polyacrylamide SDS-PAGE), which were run at a constant power of 5 watts for 45 min. Then, the power was switched to 20 watts until the bromophenol blue frontier reached the bottom of gels. 

According to standard protocols for image analysis, the gels were subsequently silver stained using 0.1% silver nitrate. To account for experimental variation, three batches of total proteins were subjected to 2D electrophoresis (2-DE), and replicate gels were simultaneously run three times. Gels were scanned using image scanner II (GE) and analyzed through Imagemaster 2D Elite version 2.00 (GE). Quantitative analysis sets were analyzed using the differences in protein spot intensity. With intensity either increased or decreased for more than twofold, protein spot was marked and confirmed through manual inspection [15].

### 2.7. In-Gel Digestion, MALDI-TOF MS, and Database Analysis

We conducted in-gel digestion of proteins from gels. Spots were destained using freshly prepared 15 mmol/L potassium ferricyanide/50 mmol/L sodium thiosulfate. Then, it was washed with 25 mmol/L ammonium bicarbonate/50% acetonitrile and dried in a SpeedVac of SC110A vacuum concentrator (Savant, Holbrook, USA). We rehydrated the dried gel pieces using 3–10 mL of 20 ng/mL trypsin solution. The solution volume was sufficient to resaturate the dried gel. Digestion was continued at 37°C for 14–18 hrs. First, we extracted tryptic peptides using 5% trifluoroacetic acid (TFA) at 40°C for 1 h, which was followed by 2.5% TFA/50% acetonitrile at 30°C for 1 h. In an Eppendorf tube, the extracted solutions were mixed and dried in a vacuum concentrator.

We solubilized the peptide mixtures using 0.5% TFA. In this case, we used saturated *α*-cyano-4-hydroxycinnamic acid (CHCA) solution, wherein 0.1% TFA/50% acetonitrile was used as the matrix. Then, we analyzed it using a Voyager DE STR MALDI-TOF mass spectrometer (ABI, Carlsbad, California, USA). We set up the following parameters: positive ion-reflector mode, accelerating voltage 20 kV, grid voltage 64.5%, mirror voltage ratio 1.12, N_2_ laser wave length 337 nm, pulse width 3 ns, the number of laser shots 50, acquisition mass range 1000–3000 Da, delay 100 ns, and vacuum degree 4 × 10^−7^ Torr. A trypsin-fragment peak served as an internal standard for mass calibration. A list of the corrected mass peaks was the PMF.

Using an MASCOT Distiller, proteins were identified using peptide mass fingerprinting (PMF) data through a searching software PeptIdent (http://www.expasy.org/) and MASCOT (http://www.matrixscience.com). It could detect peaks which attempted to fit an ideal isotopic distribution of the experimental data. The searching parameters were set up as follows: mass tolerance ±5 Da, number of missed cleavage sites allowed 1, cysteine residue modified as carbamidomethyl-cys, variable modifications oxidation (M), minimum number of matched-peptides 4, species selected as Rattus norvegicus (rat), peptide ion [M + H]^+^, mass values monoisotopic, searching range within the experimental pI value ±0.5 pH unit, and experimental Mr ± 20%. The isotope mass was used. By MASCOT searching, protein scores which were greater than 62 were considered as significant (*P* < 0.05).

### 2.8. Immunohistochemistry 

Immunohistochemical staining for atrial natriuretic factor (ANF), heat shock protein beta-6 (HSPB6), and peroxiredoxin-6 (PRDX6) was performed using routine immunohistochemistry streptavidin peroxidase method. It contained a rabbit polyclonal IgG antibody against ANF (Santa Cruz Biotechnology, sc-20158, Santa Cruz, CA, USA), PRDX6 (Santa Cruz Biotechnology, sc-134478, Santa Cruz, CA, USA), and HSPB6 (Abcam, ab13491, Cambridge, MA, USA) (1 : 100). Nuclear counterstaining was performed using hematoxylin At. Five randomly selected fields from each section were examined at a magnification of ×400 and analyzed using NIS-Elements. The positive content was calculated using the following formula: positive content (PC) = mean optical density × positive area [16].

### 2.9. Apoptosis Assay

We purchased the terminal deoxynucleotidyl transferase dUTP nick end labeling (TUNEL) apoptosis assay kit for paraffin section from Nanjing KeyGen Biotech. Inc. (Nanjing, Jiangsu, PRC). Based on the manufacturer's instructions, all the procedures were performed. Cells were defined as apoptotic if the whole nuclear area of the cell was labeled positively. The apoptotic cells and bodies were counted in 3 high-power fields. The apoptotic index (AI) was calculated as the percentage of positively staining cells: AI = number of apoptotic cells/total number of nucleated cells [17].

### 2.10. Western Blotting

Aliquots of heart tissue (40 mg) were homogenized in liquid nitrogen. It was dissolved in lysis buffer [7 mol/L urea, 2 mol/L thiourea, 4% (w/v) CHAPS, 20 mmol/L Tris, 65 mmol/L DTT, 0.2% pharmalyte 3/10 ampholyte] that was supplemented with 8 *μ*L of protease inhibitor cocktail (the protease inhibitor cocktail includes AEBSF hydrochloride 500 *μ*M, Aprotinin 150 nM, E-64 protease inhibitor 1 *μ*M, EDTA disodium 0.5 mM, and Leupetin hemisulfate 1 *μ*M. Calbiochem, San Diego, CA, USA). Then, it was centrifuged at 15,000 × g for 30 min at 4°C. Protein concentrations were determined through modified Bradford assay. The protein lysates were loaded onto 10% sodium dodecyl sulfate-polyacrylamide gel for separation. Then, it was electrotransferred to PVDF membranes and blocked in 5% nonfat milk in Tris-buffered saline (TBST, 100 mM NaCl, 50 mM Tris, 0.1% Tween-20, pH 7.5). Membranes were incubated overnight using primary antibodies [anti-ANF, anti-HSPB6, anti-Hsp20 (phospho S16) antibody (ab58522), and antiPRDX6)] at 4°C. This was followed by secondary antibodies, which were conjugated through horseradish peroxidase (HRP). We performed the chemiluminescence (ECL, GE Healthcare Bio-science, Uppsala, Sweden) detection. The images were captured and documented through a CCD system (imagestation 2000 MM, Kodak, Rochester, NY, USA). The quantitative analysis of these images was performed using Molecular Imaging Software Version 4.0, which was provided by Kodak 2000 MM System. The optical density was normalized against *β*-actin [16].

### 2.11. Caspase-3 Activity Assay

Caspase-3 activity was performed using a colorimetric activity assay kit according to the manufacturer's instructions (Beyotime Institute of Biotechnology, Haimen, Jiangsu, PRC). In brief, assays were performed by incubating 200 *μ*g protein of tissue lysate in 100 *μ*L of reaction buffer containing 5 *μ*L of caspase-3 substrate (4 mM DEVD-pNA) in 96-well plates. The reaction buffer contained 1% NP-40, 20 mM Tris-HCl (pH 7.5), 137 mM N-acetyl-cysteine, and 10% glycerol. Lysates were incubated at 37°C for 2 h. Samples were incubated in the dark and measured with a microplate reader at an absorbance of 405 nm.

### 2.12. Statistical Analysis

Each experiment was repeated at least three times.Data were represented in the form of means ± SD. All the data were analyzed using the SPSS statistical package (version 13.0, Armonk, NY, USA). Mean values were compared through one-way analysis of variance (ANOVA), analysis of variance and multicomparison was performed. Data were tested through homogeneity test for variance. If the variances were homogenous, mean values were compared through ANOVA. The differences between two groups were analyzed, based on the test of least significance difference. If the variances were not homogenous, mean values were compared using Welch's test. The differences between two groups were analyzed by Games-Howell. A value of *P* < 0.05 was considered as statistically significant.

## 3. Results

### 3.1. Effects of BYHWD on Histological and Echocardiographic Parameters

**Figure 1:**
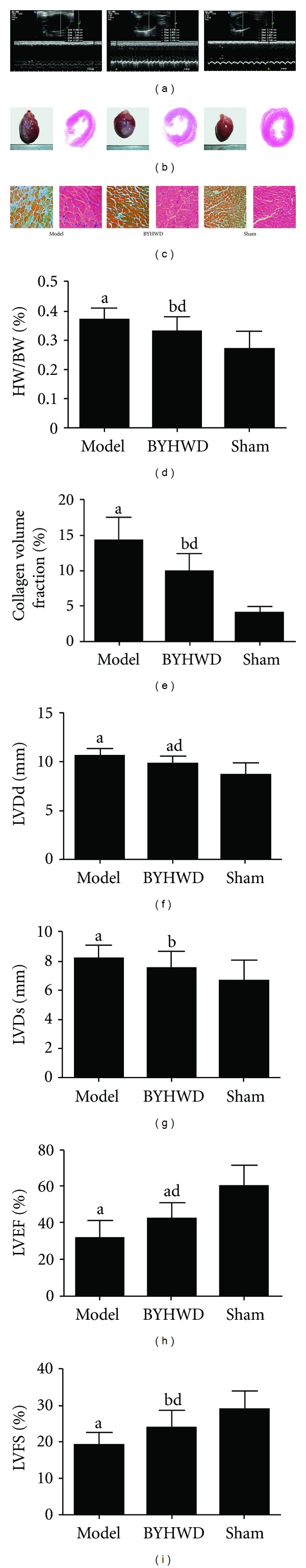
Histological and echocardiographic parameters. (a) Two-dimensional-guided M-mode echocardiographic images of the left ventricle. (b) Representative gross images of whole hearts (left), haematoxylin and eosin staining midventricular cross-sections (right). (c) Representative Masson's trichrome staining results (left) and microscopic images of haematoxylin and eosin staining (right). (d) Heart weight/body weight (HW/BW) results. (e) Collagen volume fraction results. (f)–(i) Echocardiographic measurements of left ventricular dimension at end diastole (LVDd), left ventricular dimension at end systole (LVDs), left ventricular ejection fraction (LVEF), and left ventricular fractional shortening (LVFS). (a) ^a^ *P* < 0.01, ^b^ *P* < 0.05  *versus *sham group; ^c^ *P* < 0.01, ^d^ *P* < 0.05  *versus *model group.

Compared to the sham group, a significant increase was reported in the following parameters: the heart weight/body weight (*P* < 0.01) (Figure 1(d)), left ventricular dimension at end diastole (LVDd) (*P* < 0.01) (Figure 1(f)), and left ventricular dimension at end systole (LVDs) (*P* < 0.01) (Figure 1(g)) of the model group. On the other hand, as compared to the sham group, left ventricular ejection fraction (LVEF) (*P* < 0.01) and left ventricular fractional shortening (LVFS) (*P* < 0.01) decreased significantly in the model group. The above data suggested that the ventricular remodelling model was successfully established. No significant differences were found in the following sections of the heart: left ventricular posterior wall thickness at end diastole (LVPWTd) and left ventricular posterior wall thickness at end systole (LVPWTs) (Figure 1(a)).

Observations by naked eyes showed that the heart volumes from BYHWD group were smaller than that of model group and larger than that of sham group which is consistent with the result of HW/BW ratio (Figure 1(d)). HE stain of LV showed similar trends (Figure 1(b)). Masson's trichrome staining showed that BYHWD decreased the collagen volume fraction compared to model group (*P* < 0.05) (Figures 1(c), 1(e)). BYHWD treatment decreased LVDd (*P* < 0.05) (Figure 1(f)) but not LVDs (Figure 1(g)) and increased LVEF (*P* < 0.05) (Figure 1(h)) and LVFS (*P* < 0.05) (Figure 1(i)) significantly compared to the model group.

### 3.2. Proteomics Analysis of Differentially Expressed Proteins among Model, BYHWD, and Sham Rats and Identification of Proteins with Peptide Mass Fingerprinting

**Figure 2:**
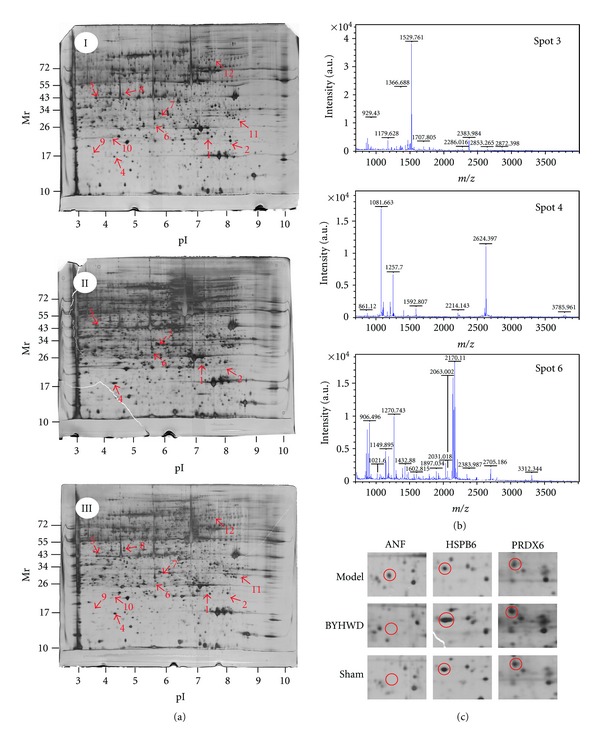
2D gel images of protein expression and MALDI-TOF MS identification of samples from area at risk in rat left ventricle. (a) 2D gel images of protein expression. Figure “I” was from model rats, Figure “II” was from BYHWD rats, and Figure “III” was from sham rats. (b) MALDI-TOF MS was obtained from spot 3, spot 4, and spot 6 after trypsin digestion. (c) Magnified images of spot 3 (ANF), spot 4 (HSPB6), and spot 6 (PRDX6).

Based on the reproducible 2D gel electrophoresis, six downregulated spots and six upregulated spots were observed in sham group as compared with the model group. Among them, three downregulated spots and four upregulated spots were observed in BYHWD-treated rats as compared with the model group. Then, they were chosen for further analysis (Figure 2(a)). All the twelve proteins were successfully identified (Table 1). Downregulated proteins, including myosin light chain 4, myosin regulatory light chain 2 (ventricular/cardiac muscle isoform), atrial natriuretic factor (ANF), and upregulated proteins, were subjected to the following conditions: heat shock protein beta-6 (HSPB6), regucalcin, peroxiredoxin-6 (PRDX6), and NADH dehydrogenase. Figure 2(b) illustrated the MALDI-TOF MS obtained from spots 3, 4, and 6 that were identified as ANF, HSPB6, and PRDX6, respectively. Figure 2(c) illustrated magnified images of ANF, HSPB6, and PRDX6, respectively.

### 3.3. Detection of the Expression of ANF, HSPB6, and PRDX6

**Figure 3:**
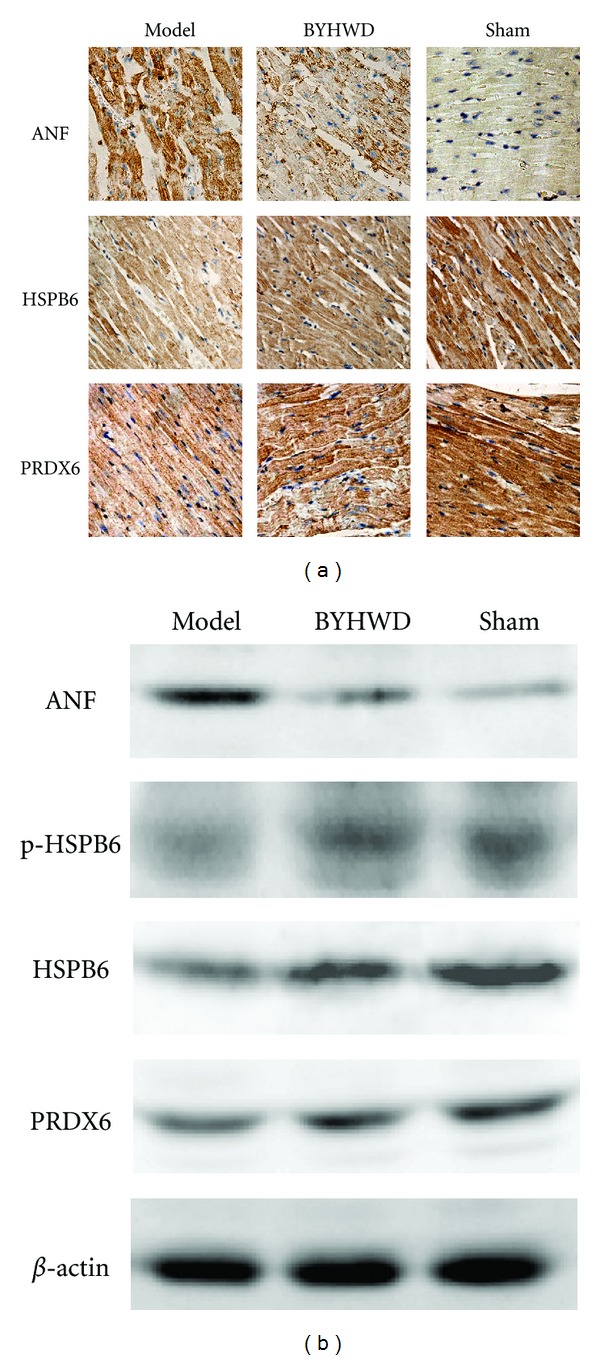
Immunohistochemical and western blotting assay of ANF, HSPB6 and PRDX6 expression in left ventricle. (a) Immunohistochemical assay of ANF, HSPB6 and PRDX6 expression. (b) Western blotting assay of ANF, HSPB6, and PRDX6 expression. Semiquantitative analysis of the above images referred to Table 2.

Immunohistochemistry (Figure 3(a)) illustrated that expression of ANF was increased and expressions of HSPB6 and PRDX6 were decreased in the model group when compared with the sham group. Western blotting (Figure 3(b)) analysis further confirmed the results of immunohistochemistry. Semiquantitative analysis of immunohistochemistry and western blotting (Table 2) showed that BYHWD treatment significantly decreased the expression of ANF (*P* < 0.01; *P* < 0.01). It also increased the expression of HSPB6 (*P* < 0.05; *P* < 0.05) and PRDX6 (*P* < 0.05; *P* < 0.05) (Table 2). Furthermore, as compared with the model group, western blotting technique suggested that BYHWD significantly increased the phosphorylation of HSPB6 (*P* < 0.05) (Figure 3(b); Table 2).

### 3.4. Effects of BYHWD Pretreatment on Myocardium Apoptosis, Expression of Bcl-2 and Bax, and Caspase 3 Activity

**Figure 4:**
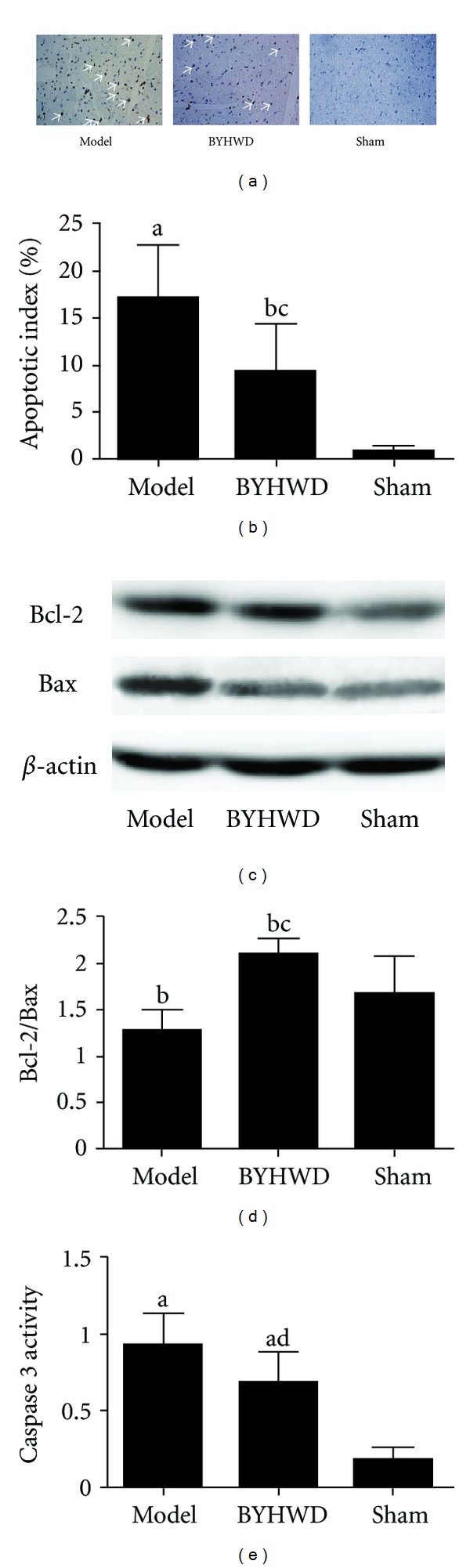
Effects of BDHWD on apoptosis and expression of Bcl-2 and Bax. (a)-(b) TUNEL analysis of apoptosis in left ventricular area at Risk. (c)-(d) Western blotting assay of Bcl-2 and Bax expression. (e) Caspase 3 activity analysis. ^a^ *P* < 0.01, ^b^ *P* < 0.05  *versus* sham group; ^c^ *P* < 0.01, ^d^ *P* < 0.05  *versus *model group.

 TUNEL staining suggested that higher number of brown stained cells were found in the model group, as compared to those of the sham group (*P* < 0.01). As compared to the model group, BDHWD significantly decreased the number of apoptotic cells (*P* < 0.01) (Figures 4(a) and 4(b)).

Western blotting technique showed that the expression of Bcl-2 and Bax (*P* < 0.01; *P* < 0.01) (Figure 4(c), Table 2) in the model group increased significantly, as compared with the sham group. As compared with the model group, BYHWD significantly decreased the expression of Bax (*P* < 0.01) but not Bcl-2. As compared with that of sham group, Bcl-2/Bax ratio of the model group decreased significantly (*P* < 0.05). As compared to that of model group, BYHWD significantly increased the ratio (*P* < 0.01) (Figure 4(d)). As compared to that of sham group, caspase 3 activity of the model group increased significantly (*P* < 0.01). As compared with that of model group, BYHWD significantly decreased caspase 3 activity (*P* < 0.05) (Figure 4(e)).

## 4. Discussion

In this study, we represent the long-term effects of BYHWD treatment in a setting of chronic MI. According to our data, the HW/BW ratio, LVDd, and LVDs in the model group were greater than those in the sham group. On the other hand, LVEF and LVFS were smaller than those in the sham group. This provides basic information on the functional changes of rats, following chronic coronary artery ligation. It also indicates the feasibility of evaluating chronic myocardial infarction induced through remodeling [18]. As compared with the model group, HW/BW and LVDd were lowered. LVEF and LVFS were raised in the BYHWD groups. These measurements were worse than those observed for sham-operated animals. Nevertheless, as compared to those in the model group with MI, most parameters in the BYHWD group were much closer to those in the sham group. BYHWD is effective in preventing ventricular remodeling, which is induced through chronic coronary artery ligation.

BYHWD decreased the expression of ANF and increased the expression of HSPB6 and PRDX6. The ablation of ANF gene was associated with a greater ratio of heart weight/body weight [19]. On the other hand, the overexpression of ANF was associated with the presence of smaller hearts [20]. In the model group, our results suggested a significant upregulation in ANF expression of the heart. This is consistent with the result that the cardiac levels of ANF are directly correlated with the degree of cardiac dysfunction of heart failure [21]. The increase of ANF which accompanied cardiac remodeling should not be interpreted as a marker of the contributory role of ANF to cardiac growth. Rather, it should be considered as a response of an intrinsic “friendly” mechanism, which compensates for heart failure [22]. So, the decrease of ANF by BYHWD responsively reduced the amelioration of ventricular dysfunction. BYHWD increases the expression and activates the phosphorylation of HSPB6 (also as heat shock protein 20, HSP20) at serine 16. An overexpression and phosphorylation of HSPB6 protects the heart against ischemia/reperfusion injury and remodeling [23, 24]. As Prdx6^−/−^ mice were more susceptible to ischemic reperfusion injury [25], it can be inferred that BYHWD ameliorates ventricular remodeling through upregulation of PRDX6. This is consistent with our previous study that elucidates the up-regulation of PRDX6. It is related with the attenuation of lipid peroxidation and inflammation [26]. 

Is there a synergistic mechanism existing among the above three protein targets to cooperate and prevent ventricular remodeling? Note that an elevation of ANP [27], blockade of HSPB6 Ser16 phosphorylation [23], and PRDX6^−/−^ mice [25] increased the apoptosis of cardiac myocytes, respectively. Our results also inferred that BYHWD ameliorated ventricular remodeling through downregulation of AI in cardiomyocytes. Some important chemical constituents of BYHWD, including Astragalus injection [28], Astragaloside IV [29] and total paeony glycoside (TPG) [30] not only improved left ventricular remodeling but also inhibited cardiac myocytes apoptosis. Herbal medicine, such as Radix Angelicae Sinensis reduced apoptosis of myocardial cells and ameliorated ventricular remodeling [31]. Ligusticum Chuanxiong prevents serum deprivation-induced PC12 cell apoptosis [32]. The extract from Danggui Buxue decoction, a subsidiary formula of BYHWD, protected the cardiomyocytes from H_2_O_2_-induced apoptosis through downregulation of caspase-3 expression [33]. Thus, the effects of these active anti-apoptotic components of BYHWD played an important role in preventing the ventricular remodeling through down regulation of AI. Furthermore, a famous “herb pair”, namely persicae Semen (Taoren) and Carthami Flos (Honghua) (TH) significantly reduced platelet aggregation and protected vascular endothelial cells [34]. Lumbrokinase, from Lumbricus, improved regional myocardial perfusion in patients diagnosed with stable angina [35] and had potential antithrombotic effects [36]. In other words, we can assume the direct protective effects of BYHWD on ischemia induced ventricular dysfunction and remodeling. This indicates that our research is related with these active constituents, especially those associated with anti-apoptotic components of BYHWD.

The above data suggested that BYHWD inhibited ischemia-induced cardiac myocyte apoptosis through Bax, Bcl-2, and caspase 3 pathways. It has been reported that both Radix Angelicae Sinensis [31] and TPG [30] activated Bcl-2 and inhibited Bax expression both at gene and protein level, then the Bcl-2/Bax ratio was increased as a matter of course. In addition, extract from Danggui Buxue decoction [33] downregulated caspase-3 in the cardiomyocytes of the rat's heart. There was a decrease of Bax expression and caspase 3 activity. It subsequently increased the Bcl-2/Bax ratio by BYHWD, which could be regulated through ANF, HSPB6, and PRDX6. There are at least two mechanisms underlying the cardioprotection of HSPB6 in apoptosis regulation. First, HSPB6 overexpression stabilizes the ischemia/reperfusion, which is induced through Bcl-2 decrease and Bax increase [37]. Second, HSPB6 preserved the integrity of mitochondria and repressed the activation of caspase-3 through complexes with Bax [37]. As a result, reduction of Bax by HSPB6 was associated with improved cardiac functions. It also reduced cardiomyocyte apoptosis by BYHWD [38].

Previous reports have suggested that peroxiredoxin II is a unique antioxidant in the cardiac system. In fact, it represented a potential target for cardiac protection, which is caused from oxidative stress-induced injury [39]. However, our results suggested that BYHWD ameliorated ischemia-induced ventricular remodeling by increasing the expression of PRDX6. The differences could be attributed to the fact that the former animal model is an acute one, whereas the latter is a chronic model. Unlike other PRDXs, PRDX6 is a bifunctional enzyme with phospholipase A2 (PLA2) activity and peroxidase function [40]. Moreover, these two functions play a pivotal role in protecting cells against oxidant stress, which is caused by an exogenous hydroperoxide [41]. Induced expression of Prdx-1 by antioxidant was accompanied with downregulation of proapoptotic gene Bax [42]. So, we inferred that PRDX mediates the protective effects of BYHWD, either through its antioxidant properties or inhibition of apoptotic signaling [43]. 

We define ventricular remodeling as ‘‘the genomic expression resulting in molecular, cellular, and interstitial changes that are manifested clinically as changes in the size, shape, and function of the heart after cardiac injury” [44]. BYHWD treatment has consistently improved hemodynamic and echocardiographic functions through several different parameters. Yet, we still cannot determine the precise mechanisms underlying these protective effects. In this study, we have identified a distinct pathway utilized by BYHWD to mediate cellular protection under ischemic conditions. The induction of HSPB6, Prdx6 and subsequent reduction of ANF are required for *in vivo* protection. Furthermore, systemic administration of BYHWD increased the Bcl-2/Bax ratio and activity of caspase 3. Accordingly, it reduced the apoptosis rate induced by ischemia through increased caspase-3 expression. This occurred in association with the heart failure and apoptosis of experimental animals [45]. Taken together, these data based on proteomic analysis suggests that BYHWD ameliorated ventricular remodeling through multiple-apoptosis-related signal pathways that converged on HSPB6, Prdx6, and ANF to decrease Bax and caspase 3 activities. Further studies were conducted to investigate the direct relationship between the function of BYHWD and HSBP6, Prdx6. Moreover, their regulation on apoptosis will be instrumental in defining novel therapeutic approaches to ischemic remodeling injury.

## Supplementary Material

Reproducibility of BYHWD sample by HPLC analysis.

## Figures and Tables

**Table 1:** MALDI-TOF MS identification of protein molecules with altered expression.

Spot no.^ a^	Accession no.	Protein score (threshold)^b^	Molecular weight^c^	Calculated pI value^c^	Protein name	Expression level^d^
1	P17209 MYL4_RAT	161 (23)	21282.18	4.96	Myosin light chain 4	↓, ⇓
2	Q08733 MLRV_RAT	174 (34)	18880.35	4.86	Myosin regulatory light chain 2, ventricular/cardiac muscle isoform	↓, ⇓
3	01161 ANF_RAT	132 (34)	16555.55	6.73	Atrial natriuretic factor	↓, ⇓
4	P97541 HSPB6_RAT	82 (24)	17504.93	6.05	Heat shock protein beta-6	↑, ⇑
5	Q03336 RGN_RAT	75 (23)	33389.85	5.27	Regucalcin	↑, ⇑
6	O35244 PRDX6_RAT	66 (23)	24818.6	5.64	Peroxiredoxin-6	↑, ⇑
7	Q561S0 NDUAA_RAT	80 (23)	40493.09	7.64	NADH dehydrogenase	↑, ⇑
8	Q63347 PRS7_RAT	46 (27)	48574.78	5.59	26S protease regulatory subunit	↑
9	P61983 (1433G_RAT)	64 (27)	28302.59	4.80	14-3-3 protein gamma	↓
10	P02091 (HBB1_RAT)	73 (23)	15979.39	7.87	Hemoglobin subunit beta-1	↑
11	O35796 (C1QBP_RAT)	43 (23)	30996.92	4.77	Complement component 1 Q subcomponent-binding protein, mitochondrial	↓
12	P11884 (ALDH2_RAT)	49 (23)	56488.42	6.63	Aldehyde dehydrogenase, mitochondrial	↓

Notes: ^a^Defined according to spot positions in 2-D gel indicated as in Figure 2.

^b^Protein scores are derived from ions scores as a nonprobabilistic basis for ranking protein hits. Protein scores greater than threshold value are significant (*P* < 0.05).

^c^Molecular weight value calculated by amino acid count; pI value calculated from the database entry without any processing.

^d^ ↑↓Expression of sham group compared to that of model group; ⇑⇓ expression of BDHWD group compared to that of model group.

**Table 2:** Semiquantitive analysis of ANF, HSPB6, and PRDX6 by IHC and WB.

	Model	BYHWD	Sham
ANF-IHC	5.09 ± 1.59^a^	3.07 ± 0.92^ac^	1.47 ± 0.68
HSPB6-IHC	5.67 ± 1.39^a^	7.48 ± 1.57^ad^	9.91 ± 2.62
PRDX6-IHC	4.44 ± 1.59^a^	6.39 ± 2.11^bd^	8.94 ± 2.54
ANF-WB	0.33 ± 0.06^a^	0.13 ± 0.04^bc^	0.04 ± 0.02
pHSPB6-WB	0.02 ± 0.01^a^	0.07 ± 0.02^bd^	0.11 ± 0.03
HSPB6-WB	0.23 ± 0.04^a^	0.39 ± 0.04^bd^	0.53 ± 0.08
PRDX6-WB	0.10 ± 0.01^a^	0.16 ± 0.03^ad^	0.26 ± 0.03
Bax	0.33 ± 0.04^a^	0.28 ± 0.03^c^	0.17 ± 0.02
Bcl-2	0.26 ± 0.03^a^	0.14 ± 0.03^a^	0.11 ± 0.02

IHC: immunohistochemistry; WB: western blotting.

^a^ *P* < 0.01, ^b^ *P* < 0.05*  versus* sham group; ^c^ *P* < 0.01, ^d^ *P*<0.05 *versus* model group.
