# Supplementary material for: Effects of Buyang Huanwu Decoction on Ventricular Remodeling and Differential Protein Profile in a Rat Model of Myocardial Infarction
Source: Evid Based Complement Alternat Med. 2012 Sep 19;2012:385247. doi: 10.1155/2012/385247 (PMC3459299; doi:10.1155/2012/385247)
Supplement: Supplementary file 1 — Reproducibility of BYHWD sample by HPLC analysis. [file 385247.f1.doc]

**Supplementary material**

The high performance liquid chromatography (HPLC) analysis performed simultaneous analyses using a Shimadzu LC-20A HPLC system (Shimadzu Co., Kyoto, Japan), comprising a solvent delivery unit, a non-line degasser, a column oven, an auto-sampler, and a Photodiodearraydetector (PDA) detector.

The data processor used LC solution software. The analytical column includes a Shimadzu C_18_ column (250×4.6 mm; particle size 5 mm; Japan). The mobile phases comprised water (A) and methanol (B). The gradient flow was as follows: 0–60 min, 10%–95% B; 60–65 min, 95% B; 65–70 min, 95%–10% B; 70–80 min, 10% B. The analysis was performed at a flow rate of 1.0 mL/min with PDA detection at 225 nm. The injection volume was 20 μL.

The Similarity Evaluation System for Chromatographic Fingerprint of TCM (2004 A edition) was used to evaluate the similarities of the 6 batches of BYHWD. After peak-picking, template-matching process, the peaks in the spectra were matched automatically (Fig. S1). The reference template was set finally for spectra peak difference and entire similarity evaluation. The similarities of repeatability were from 0.989 to 0.999 (Table S1)．The results showed that the preparation process of BYHWD was reasonable; the quality of BYHWD could be cotrolled.


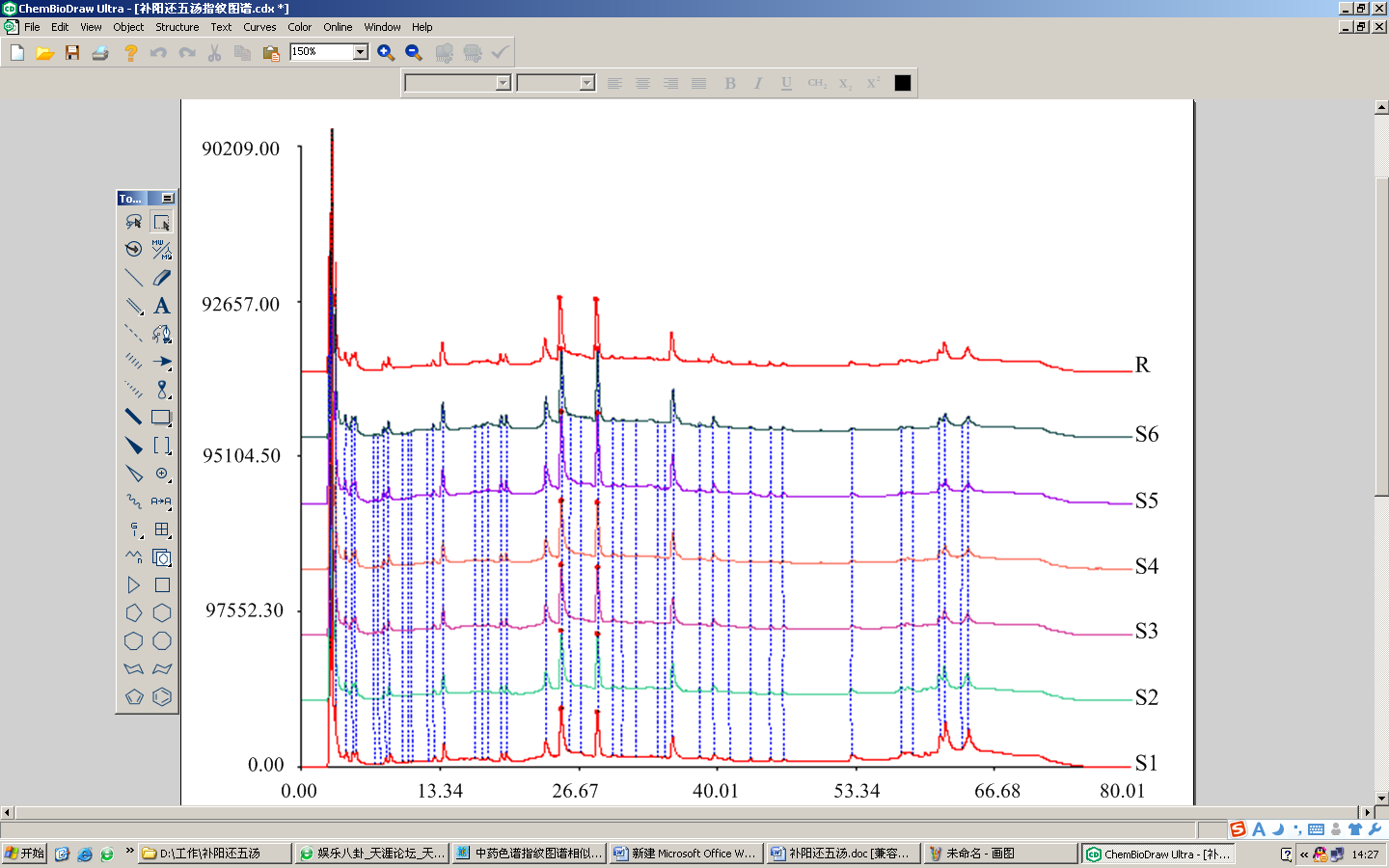


Fig. S1 The matched chromatographys of 6 batches of BYHWD samples

Table S1. Comparability result of reproducibility of BYHWD samples

|  | Sample 1 | Sample 2 | Sample 3 | Sample 4 | Sample 5 | Sample 6 | Reference Fingerprint |
| --- | --- | --- | --- | --- | --- | --- | --- |
| Sample 1 | 1.000 | 0.994 | 0.993 | 0.992 | 0.989 | 0.989 | 0.994 |
| Sample 2 | 0.994 | 1.000 | 0.999 | 0.999 | 0.995 | 0.996 | 0.999 |
| Sample 3 | 0.993 | 0.999 | 1.000 | 1.000 | 0.998 | 0.998 | 1.000 |
| Sample 4 | 0.992 | 0.999 | 1.000 | 1.000 | 0.998 | 0.998 | 0.999 |
| Sample 5 | 0.989 | 0.995 | 0.998 | 0.998 | 1.000 | 1.000 | 0.999 |
| Sample 6 | 0.989 | 0.996 | 0.998 | 0.998 | 1.000 | 1.000 | 0.999 |
| Reference Fingerprint | 0.994 | 0.999 | 1.000 | 0.999 | 0.999 | 0.999 | 1.000 |
